# Oral microbial community typing of caries and pigment in primary dentition

**DOI:** 10.1186/s12864-016-2891-z

**Published:** 2016-08-05

**Authors:** Yanhui Li, Cheng-Gang Zou, Yu Fu, Yanhong Li, Qing Zhou, Bo Liu, Zhigang Zhang, Juan Liu

**Affiliations:** 1 Department of Preventive & Pediatric Dentistry, The Affiliated Stomatology Hospital of Kunming Medical University, Kunming, Yunnan 650100 China; 2 State Key Laboratory for Conservation and Utilization of Bio-Resources in Yunnan, Yunnan University, Kunming, Yunnan 650091 China; 3 Department of Dermatology, Beijing Hospital, Beijing, 100730 China; 4 State Key Laboratory of Genetic Resources and Evolution, Kunming Institute of Zoology, Chinese Academy of Sciences, Kunming, 650223 China

**Keywords:** Saliva, Supragingival plaque, Pigment, Caries, Oral microbiota

## Abstract

**Background:**

Black extrinsic discoloration in primary dentition is a common clinical and aesthetic problem that can co-occur with dental caries, the most common oral diseases in childhood. Although the role of bacteria in the formation of pigment and caries in primary dentition is important, their basic features still remain a further mystery.

**Methods:**

Using targeted sequencing of the V1-V3 hypervariable regions of bacterial 16S ribosomal RNA (rRNA) genes, we obtained a dataset consisting of 831,381 sequences from 111 saliva samples and 110 supragingival plaque samples from 40 patients with pigment (black extrinsic stain), 20 with caries (obvious decay), and 25 with both pigment and caries and from 26 healthy individuals. We applied a Dirichlet multinomial mixture (DMM)-based community typing approach to investigate oral microbial community types.

**Results:**

Our results revealed significant structural segregation of microbial communities, as indicated by the identification of two plaque community types (A and B) and three saliva community types (C-E). We found that the independent occurrence of the two plaque community types, A and B, was potentially associated with our oral diseases of interest. For type A, three co-occurring bacterial genus pairs could separately play a potential role in the formation of pigment (*Leptotrichia* and *Fusobacterium*), caries (unclassified *Gemellales* and *Granulicatella*), and mixed caries and pigment (*Streptococcus* and *Mogibacterium*). For type B, three co-occurring bacterial genera (unclassified *Clostridiaceae, Peptostreptococcus*, and *Clostridium*) were related to mixed pigment and caries. Three dominant bacterial genera (*Selenomonas, Gemella,* and *Streptobacillus*) were linked to the presence of caries.

**Conclusions:**

Our study demonstrates that plaque-associated oral microbial communities could majorly contribute to the formation of pigment and caries in primary dentition and suggests potential clinical applications of monitoring oral microbiota as an indicator for disease diagnosis and prognosis.

**Electronic supplementary material:**

The online version of this article (doi:10.1186/s12864-016-2891-z) contains supplementary material, which is available to authorized users.

## Background

Discovering the dynamics and relationships of human oral microbial community types will allow microbiologists to better associate changes in the microbiome with changes in health or disease. It is difficult to differentiate disease-associated microbiome changes from other factors by limiting inter-individual variation to the minimum amount, although the partitioning around meloids (PAM)-based approach can capture various human gut microbial enterotypes [1]. Recently, Ding & Schloss employed an novel approach based on the Dirichlet multinomial mixture (DMM) model independent of the distance matrix [2]; this method significantly improved the microbial community typing across the human body that was performed by analyzing 16S ribosomal RNA (16S rRNA) gene sequences [3]. For example, empirical comparison of the methods indicated that only two community types were identified in samples of saliva and supragingival plaque using the PAM-based approach, whereas four and six community types with the lowest Laplace values (for selecting the best number of metacommunities) were separately identified at the two oral sites using the DMM-based approach. These findings suggest that the DMM-based approach is superior for assessing the oral microbial community types associated with oral diseases, such as caries and pigment.

Caries is one of the most common oral diseases in childhood, and it results from many factors, including bacterial infection [4]. Recent studies have demonstrated that altered oral microbiota may be associated with caries [5–7]. However, oral microbial communities vary in a spatiotemporal manner among populations [8, 9]. Thus, the microbial configurations associated with caries, a polymicrobial disease [10], during the same dentition stage remain poorly understood.

Black extrinsic discoloration in primary dentition is another common clinical and aesthetic problem in childhood that can co-occur with dental caries [11–15]. Black stain is considered a form of dental plaque that differs from other types by insoluble iron salts and high calcium and phosphate contents [16–18]. In particular, an association between black tooth stain and bacteria, such as actinomyces, has been reported [19–22]. However, how microbial assemblies in both saliva and plaque are linked to pigment (represented by black extrinsic stain) and to the presence of a combination of pigment and caries remains largely unknown.

To understand the role of bacteria in pigment (black extrinsic stain) formation and its co-occurrence with caries, we performed a large-scale molecular analysis of 16S rDNA sequences using a DMM-based community typing approach to address two crucial issues: whether there are significant divergences of microbial communities associated with caries and/pigment within specific oral niches and what are the dominant constituents that potentially contribute to the occurrence of caries, pigment and their combination?

## Methods

### Patient selection and sample collection

Through visual inspection by a single investigator (Y.HG.L.), a specialist in pediatric dentistry, the status of caries was determined according to the International Caries Detection and Assessment System (ICDAS-II) [23], and black extrinsic tooth stain was evaluated based on the presence of pigmented dark lines parallel to the gingival margin or an incomplete coalescence of dark dots rarely extending beyond the cervical third of the crown [16, 24]. The health status of the volunteers was self-reported. The exclusion criteria were as follows: 1) having oral diseases, such as periodontitis, salivary gland disorders, and oral mucosal diseases; 2) having autoimmune disease; 3) receiving any antibiotic treatment in the past three months; 4) a history of smoking history; 5) drinking tea, coffee, and other beverages with tannin in the past month; and 6) having surgical procedures during the several years prior to this study. In total, 26 healthy subjects without caries and pigment, 40 patients with pigment (black extrinsic stain) (Additional file 1: Figure S1), 20 patients with caries (obvious decay), and 25 patients with both pigment (black extrinsic stain) and caries (obvious decay) in their primary dentition were recruited to donate samples on March 1, 2011 in Kunming, China. Multiple supragingival plaques were removed aseptically and pooled together from each patient or healthy subject. At the same time, saliva from each patient or healthy individual was extracted using 2-ml sterile needle tubing. All salivary and supragingival plaque samples were placed in cryovials without preservative, immediately snap frozen in liquid nitrogen, and stored at −80 °C until bacterial community analysis.

### Saliva and supragingival plaque sample preparation and DNA extraction

Each sample was suspended while frozen in a solution containing 200 μl of buffer ATL and 200 μl of a slurry of 0.1-mm diameter zirconia/silica beads (BioSpec Products, Bartlesville, OK). The mixed sample was then lysed by mechanical disruption with a bead beater (BioSpec Products) set on high for 2 min (20 °C), followed by extraction with a QIAamp® DNA Stool Mini Kit (Qiagen, Hilden, Germany) according to its protocol for the isolation of DNA for pathogen detection. The DNA from each sample was eluted in a final volume of 200 μl of elution buffer and stored at −20 °C.

### PCR amplification of the V1-V3 region of bacterial 16S rRNA genes

Triplicate PCR reactions were performed for each sample. We referred previous studies [25, 26] for primer designs as follows: the forward primer (5′-CGTATCGCCTCCCTCGCGCCATCAGNNNNNNN*AGAGTTTGATCMTGGCTCAG*-3′) contained the *454 Life Sciences* primer A sequence, a unique 7-nt barcode used to tag each PCR product (designated by NNNNNNN) and the broad-range bacterial primer 8 F. The reverse primer (5′-CTATGCGCCTTGCCAGCCCGCTCAG*GTATTACCGCGGCTGCTGGCAC*-3′) contained the *454 Life Sciences* primer B sequence and the broad-range bacterial primer 533R. Each 25-μl reaction contained 0.2 μM forward and reverse primers, 3 μl of template DNA, 2.5 μl of 10× PCR buffer plus Mg^2+^ (TaKaRa, Dalian, China), 2.0 μl of dNTP (2.5 mM each) (TaKaRa), 0.75 μl of DMSO (100 %), and 0.25 μl of *TaKaRa Taq*^*TM*^ (5 U/μl). All dilutions were performed using sterile ddH_2_O. The PCR reactants were assembled in a PCR hood in which all surfaces and pipettes had been decontaminated by 30 min of autoclaving at 120 °C and 30 min of UV irradiation. Thermal cycling took place at 94 °C for 3 min, followed by 5 cycles of 94 °C for 20 s, 45 °C for 20 s, and 65 °C for 60 s and then 20 cycles of 94 °C for 30 s, 58 °C for 20 s, and 72 °C for 30 s, with a final extension at 72 °C for 5 min. Replicate amplicons were pooled and visualized on 1.5 % agarose gels using EB stain in 0.5X TE. Amplicons were cleaned using a MinElute® Gel Extraction Kit (Qiagen, Shanghai, China) according to the manufacturer’s instructions. Next, we performed pyrosequencing using primer A and titanium chemistry on a *454 Life Sciences* Genome Sequencer FLX instrument (*Roche*) at Sangon Biotech (Shanghai) Co., Ltd, China.

### Sequence data preparation and preliminary analysis

Sequences were processed and analyzed using the Qiime pipeline [27]. After quality control with a minimum quality score of Q20 per base, the quality control sequence dataset was clustered into operational taxonomic units (OTUs) using denovo usearch61 [v6.1.544 (beta)] [28] with the criterion of a minimum identity of 97 %. The taxonomy of the OTUs was determined using BLAST (E-value = 0.001) against the Greengenes database from the August 2013 release [29]. Based on the identified OTU dataset and the corresponding taxonomic assignments, we constructed an OTU count table with the taxonomic information. We excluded singletons (one sequence per OTU) from the OTU table when performing the following analyses. Based on an rarefaction analysis, all samples were sub-sampled to the same read depth to limit the effects of differential sampling that are known to affect alpha and beta diversity metrics and to differentially increase the representation of PCR and sequencing artifacts in datasets. Finally, we created a table of counts for the number of times each OTU or genus-level phylotype was observed in each sample. Principal Coordinate Analysis (PCoA) in this study is used to compare groups of samples based on both unweighted *Unifrac* [30] and binary jaccard distance metrics.

### Community typing

The resulting table was used as an input to partition all samples according to community types using the DMM [2, 3] and PAM [31] models supplied in mothur version 1.36.1 [32]. The analysis was performed at least ten times to confirm that we had obtained the minimum Laplace approximation used as the criteria for selecting the number of community types [3]. Samples were assigned to their community type based on the maximum posterior probability. Community types were visualized by non-metric dimensional scaling (NMDS) ordination of Bray-Curtis divergence values between oral samples using the DMM approach in mothur.

### Statistics

General characteristics were represented as the mean or median. A hierarchically clustered heatmap analysis of oral samples was performed based on microbial abundances using average linkage with *Pearson* correlation, using the function aheatmap in Nonnegative Matrix Factorization (NMF) *R* package [33]. Multiple sample comparisons were performed using one-way analysis of variance (ANOVA) (parametric) or Kruskal-Wallis one-way ANOVA on ranks (non-parametric) using SigmaPlot 12.0 (Systat Software, Inc.). Based on the Bray-Curtis distance, the analysis of molecular variance (AMOVA) (determines whether the genetic diversity within two or more communities is greater than their pooled genetic diversity) and homogeneity of molecular variance (HOMOVA) (determines whether the amount of genetic diversity in each community is significantly different) provided by mothur version 1.36.1 [32] were used to compare the community structures. Co-occurring analysis using the Spearman rank correlation was conducted using Hmisc 3.9–3 (Harrell, Vanderbilt University School of Medicine, Nashville, TN, USA) in the R software package and the relative abundance of bacterial genera. Each co-occurring pair had an absolute value of Spearman rank correlation coefficient (r) > 0.50 with statistical significance level under 0.0001.

## Results

We collected 111 saliva and 110 supragingival plaque samples from the primary dentition of 85 patients and 26 healthy subjects, yielding a total of 221 samples for molecular analysis of the bacterial 16S rRNA genes. We obtained a dataset consisting of 831,381 high-quality 16S rRNA gene sequences (the V1-V3 hypervariable regions) with an average of 3,762 ± 165 (S.E.) sequences per sample. The PCoA analyses based on both unweighted *Unifrac* and binary jaccard measures consistently showed significant (*P* = 0.001, anosim test) segregation of salivary and plaque samples (Additional file 2: Figure S2A). To limit the effects of differential sampling and to render the microbial communities of samples comparable, we performed a rarefaction curve analysis (Additional file 3: Figure S3) by which we found that 2000 sequence sub-sampling was adequate to reflect the complex of oral microbiota because the increase of observed species tends to slow. Thus, to compare samples, all samples were sub-sampled to 2,000 reads following the removal of 23 salivary and 20 plaque samples with reads fewer than 2,000. The final 178 samples were used to create a table of counts for each OTU (or species)-level phylotype observed in each sample for PCoA-based community typing. The result still reflected significantly (*P* = 0.001, anosim test) microbial community divergence dependent on the heterogeneity of salivary and plaque niches (Additional file 2: Figure S2B).

**Fig. 1:**
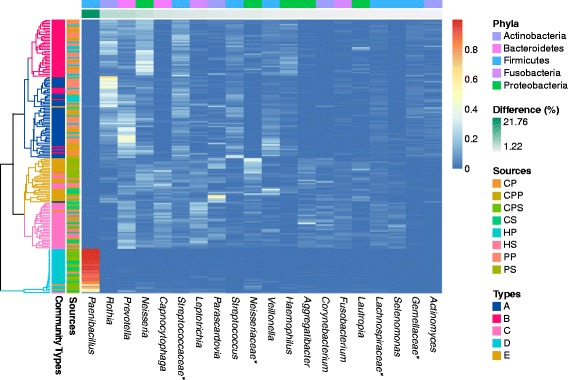
178 samples were hierarchically clustered into five groups based on oral bacterial genera using average linkage with *Pearson* correlation. The heat map shows relative abundance of top 20 bacterial genera (horizontal axis) in each sample (vertical axis) contributing to 72.55 % of the total difference for assessing five community types with DMM approach. The stars denote unclassified taxa. HP: Healthy Plaque; CP: Caries-active Plaque; PP: Pigment-occurred Plaque; CPP: Caries + Pigment Plaque; HS: Healthy Saliva; CS: Caries-active Saliva; PS: Pigment-occurred Saliva; CPS: Caries + Pigment Saliva

**Fig. 2:**
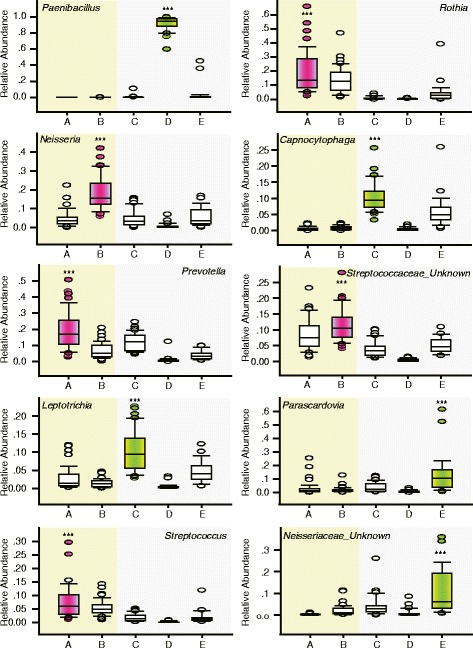
Top ten bacterial genera contributing to community types. The labels located along the horizontal axis of each panel represent five oral microbial community types (A and B belong to plaque samples, and C-E belong to saliva samples). The relative abundance of the ten most abundant genera in the samples assigned to each of the types (the boxes represent the interquartile range (IQR), and the error bars represent the 95 % confidence intervals; *n* (community type A) = 45; *n* (community type B) = 45; *n* (community type C) = 37; *n* (community type D) = 26; *n* (community type E) = 25). Statistical significance was evaluated using the Kruskal-Wallis one-way analysis of variance on ranks. *P*-values: *** < 0.001, ** < 0.01, and * < 0.05

To discover disease-associated community-level changes of the oral microbial world, we performed DMM-based community typing based on genus-level phylotype observed in each of the 178 samples. We identified five community types supported by the smallest Laplace value after ten independent runs; these communities included two types (A and B) in plaque niches and three types (C, D, and E) in saliva, which was visualized by NMDS method (Additional file 4: Figure S4). Between 91 % and 100 % of the samples assigned into five community types had a posterior probability of at least 0.99. Significant differences among and within five communities were further confirmed by both HOMOVA and AMOVA tests (*P* < 0.001). Furthermore, we found 20 genus-level taxa contributing to 72.55 % of the total difference for assessing five community types with DMM approach (Fig. 1). All 178 samples were hierarchically clustered into similar five community groups based on relative abundances of those 20 genera (Fig. 1). We further compared the differences of the top ten genera contributing to 56.9 % of the total differences among five community types (Fig. 2). The results found each of five types dominated by specific bacterial compositions: *Rothia, Prevotella,* and *Streptococcus* were the most dominant (*P* < 0.001, Kruskal-Wallis one-way ANOVA on ranks) in type A, *Neisseria* and unclassified *Streptococcaceae* (*P* < 0.001) in type B, *Leptotrichia* (*P* < 0.001) in type C, *Paenibacillus* in type D, and *Parascardovia* and unclassified *Neisseriaceae* in type E (*P* < 0.001).

**Fig. 3:**
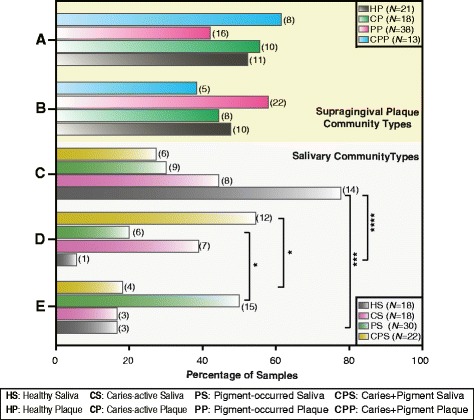
Sample distribution comparisons across community types. The labels located along the vertical axis represent five oral microbial community types (**a** and **b** belong to plaque samples, and **c**-**e** belong to saliva samples). Each bar represents the sample distribution percentage from different sources in each community type within the same niches. Sample number of each source assigned to each type placed to the right of the bar. Statistical significance was evaluated using Fisher’s test. *P*-values: **** < 0.0001, *** < 0.0001, ** < 0.01, and * < 0.05

To identify the oral microbial community types potentially associated with diseases, we compared sample distribution differences among community types within plaque or salivary niches (Fig. 3). First, there was no significant difference in the samples of the same sources between the two plaque community types, A and B. Second, for the three salivary community types (C-E), we found that for the salivary samples of healthy subjects, type C was significantly more likely than type D (*P* < 0.0001, Fisher test) and type E (*P* < 0.001), suggesting that type C may be representative of healthy individuals (Fig. 3). In addition, for salivary samples of patients with mixed caries and pigment (CPS), type D was significantly (*P* < 0.05) more likely than type E, whereas for the salivary samples of patients who only had pigment (PS), type E was significantly (*P* < 0.05) more likely than in type D (Fig. 3). Such marginal statistic significances weakly supported the divergence of two salivary community types (D and E) likely associated with the presence of CPS and PS.

**Fig. 4:**
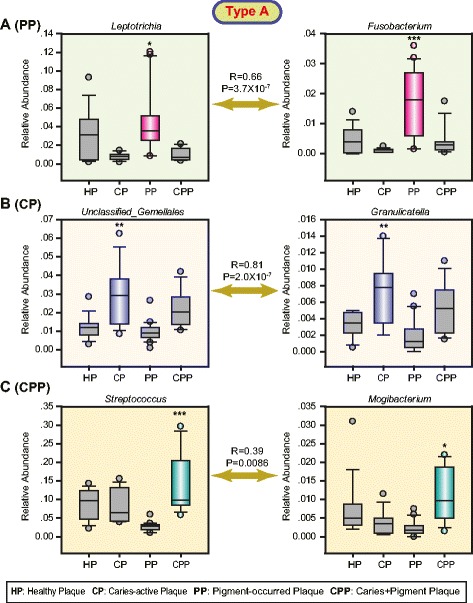
Dominant bacterial genera are potentially associated with the presence of pigment (**a**), caries (**b**), and mixed caries/pigment (**c**) in the plaque-unique community type A. The relative abundance of the most abundant genera in the samples assigned to each of the sample types (the boxes represent the IQR, and the error bars represent the 95 % confidence interval; *n* (HP) = 11; *n* (CP) = 10; *n* (PP) = 16; *n* (CPP) = 8). Statistical significance was evaluated using the Kruskal-Wallis one-way analysis of variance on ranks with adjusted *P*-values: *** < 0.001, ** < 0.01, and * < 0.05. The pairwise positive correlations marked by double-arrow lines were calculated using the Spearman rank-order correlation with the correlation coefficient *R* and the *P*-values

**Fig. 5:**
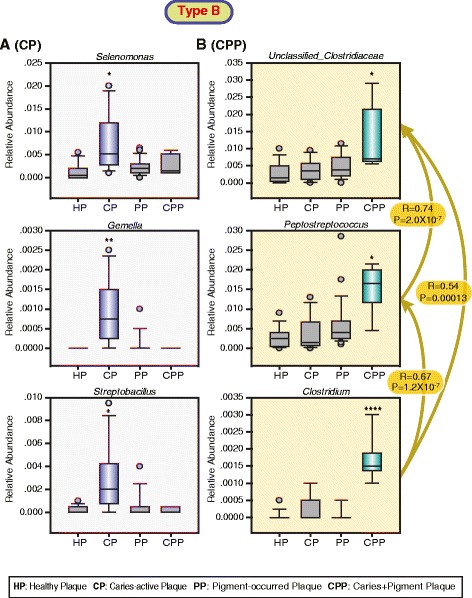
Dominant bacterial genera are potentially associated with the presence of caries (**a**) and mixed caries/pigment (**b**) in the plaque-unique community type B. The relative abundance of the most abundant genera in the samples assigned to each of the sample types (the boxes represent the IQR, and the error bars represent the 95 % confidence interval; *n* (HP) = 10; *n* (CP) = 8; *n* (PP) = 22; *n* (CPP) = 5). Statistical significance was evaluated using the Kruskal-Wallis one-way analysis of variance on ranks with adjusted *P*-values: *** < 0.001, ** < 0.01, and * < 0.05. The pairwise positive correlations marked by lines with an arrow were calculated using the Spearman rank-order correlation with the correlation coefficient R and the P values

**Fig. 6:**
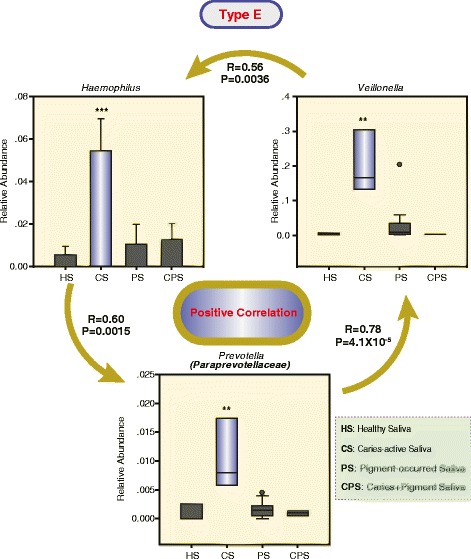
Dominant bacterial genera are potentially associated with the presence of caries in the saliva-unique community type E. The relative abundance of the most abundant genera in the samples assigned to each of the sample types (the value for the genus *Haemophilus* is expressed as the mean ± s.d.; the other two genera are shown with the IQR and the 95 % confidence interval; *n* (HS) = 3; *n* (CS) = 3; *n* (PS) = 15; *n* (CPS) = 4). Statistical significance was evaluated using Student’s t-test (the genus *Haemophilus*) or the Kruskal-Wallis one-way analysis of variance on ranks (the other two genera) with adjusted *P*-values: *** < 0.001, ** < 0.01, and * < 0.05. The positive correlations among three genera marked by lines with arrows were calculated using the Spearman rank-order correlation with the correlation coefficient R and the *P* values

To further characterize disease-associated community types, we determined the dominant bacterial genera potentially associated with diseases from five community types. For type A (Fig. 4), three significantly and positively correlated bacterial genus pairs were separately identified in pigment-containing plaques (PP) (*Leptotrichia* and *Fusobacterium*), caries-active plaques (CP) (unclassified *Gemellales* and *Granulicatella*), and plaques with the occurrence of mixed caries and pigment (CPP) (*Streptococcus* and *Mogibacterium*). All these bacterial genera were significantly (*P* < 0.05–0.001, Kruskal-Wallis one-way ANOVA on ranks) dominant in three disease types PP, CP, and CPP, respectively. For type B (Fig. 5), three dominant (*P* < 0.05–0.01, Kruskal-Wallis one-way ANOVA on ranks) bacterial genera (unclassified *Clostridiaceae, Peptostreptococcus*, and *Clostridium*) were significantly and positively correlated in CPP. Three dominant (*P* < 0.05–0.0001, Kruskal-Wallis one-way ANOVA on ranks) bacterial genera (*Selenomonas, Gemella,* and *Streptobacillus*) were identified in CP but were not correlated. In addition, we did not find any difference for two salivary types C and D in the bacterial composition across healthy subjects and patients. Consistent with those observations, HOMOVA test analysis found that those two community types had the minimum community diversity variations. For type E, we found that CS was dominated (*P* < 0.01–0.001, Kruskal-Wallis one-way ANOVA on ranks) by three significantly and positively correlated bacterial genera (*Haemophilus*, *Veillonella,* and *Prevotella* in the Paraprevotellaceae family) (Fig. 6). Totally, these findings suggested that two plaque type A and B as well as salivary type E may be associated with our concerned three disease types, although the lack of strong evidences from sample distributions across community types within salivary or plaque niches.

## Discussion

For oral microbial community typing, we found that genus-based community typing (five types identified) using the DMM approach was superior to OTU-based analysis (two types inferred by PCoA in Additional file 2: Figures S2; three types by DMM in Additional file 4: Figure S4). Because our identified five community types not only reflected microbial community divergences driven by the heterogeneity of oral environmental niches (*e.g.*, saliva and supragingival plaque), but also showed potential disease-associated community-level changes of oral microbiota within the same niche, such as two community types upon plaque. These results suggest that oral microbial diversity at the genus level can provide high-resolution information regarding the potential role of microbial infections in caries and pigment. We also found that both two plaque types and one salivary type dominated by specific bacterial genera may be associated with the occurrence of caries and pigment. Thus, further large-scale investigations combined with metadata describing changes in health or lifestyle would be interesting to reinforce those findings, because oral microbial diversities are greatly influenced by both infectious agents and other complex host factors, including lifestyle, genotype, physiology and the immune system [34]. Therefore, it is critical to use a statistically sound measurement of oral microbial diversity to distinguish infectious agents from other host factors.

Multiple distinct oral bacterial components between healthy subjects and patients were discovered from each plaque community type associated with our diseases of interest (Figs. 4 and 5). Conversely, only three correlated bacterial genera in saliva were found to be associated with caries (Fig. 6). These results suggest that plaque-associated microbial communities are much more effective than salivary microbial communities as microbial biomarkers for diagnosing and/or preventing caries and pigment. For instance, bacterial species belonging to *Veillonella*, *Actinomyces*, and *Granulicatella* are frequently found to contribute to dental caries [7, 35]. Consistently, our study demonstrated that a positive interplay (*R* = 0.81, *P* = 2.0 × 10^−7^) between *Granulicatella* and unclassified *Gemellales* in plaque-community type A (Fig. 4) was closely related to the occurrence of caries. These findings tend to support the polymicrobial nature of oral infectious diseases [36]. In addition, from plaque-community type B, the independent overgrowth of *Selenomonas*, *Gemella*, and *Streptobacillus* was observed in caries-active patients (Fig. 5a). Of them, *Selenomonas* species were found to be common in advanced caries [37]. *Gemella* species have been isolated and observed experimentally to have high degrees of autoaggregation associated with the initiation and development of dental plaque and biofilms [38]. Recently, a *Streptobacillus* species has also been found to be a novel plaque bacterial phylotype in children younger than 30 months with caries [7]. Taken together, these findings suggest that plaque-associated infectious agents might play a critical role in the formation of caries in primary dentition. Notably, saliva-associated *Veillonella* was significantly more abundant in caries-active patients than in healthy subjects (Fig. 6). *Haemophilus* and *Prevotella* (*Paraprevotellaceae*) demonstrated consistent overgrowth with *Veillonella.* One previous study has confirmed that the central role of *Veillonella sp.* in multispecies community formation is a key element in facilitating the succession of species during the development of dental plaque in vivo [39]. These results suggest that the overgrowth of *Veillonella* species in saliva may indicate a high risk of dental caries in primary dentition.

Another important finding is that the co-overgrowth of *Leptotrichia* and *Fusobacterium* belonging to the bacterial phylum Fusobacteria in plaque might contribute to the formation of pigment in primary dentition (Fig. 4A). Correspondingly, a high 29.4 % detection rate for *Leptotrichia* species was reported in dental plaque samples from patients with black extrinsic tooth staining [40]. *Fusobacterium* has also been identified in black stain samples [19]. *F. periodontium*, which was first isolated from advanced periodontitis lesions [41], is an opportunistic pathogenic bacterium of the mouth and other body sites [42]. Genome evidences from the human oral *L. buccalis* [43] demonstrate that this bacterium can express genes involved in producing beta-Lactam resistance (see the KEGG pathway map: http://www.genome.jp/dbget-bin/www_bget?lba:Lebu_0334), which could protect other penicillin-susceptible bacteria from penicillin by producing the enzyme beta-lactamase, such as *Fusobacterium spp*. [44]. These findings indicated that *L. buccalis* may promote the oral colonization of *Fusobacterium spp.* for further infection. *Fusobacterium spp*. can co-aggregate with black-pigmented anaerobes (*Porphyromonas gingivalis* and *Prevotella nigrescens*) [45]. Similarly, co-infecting or co-culturing *Pseudomonas aeruginosa* can induce pigment production of *Staphylococcus aureus* [46]. These observations provide an important implication into understanding potential role of complex bacterial interactions in the formation of pigment. Thus, it is greatly expected to confirm these findings via future in vivo or in vitro studies. In addition, the co-occurrence of *Leptotrichia* and *Fusobacterium* was only observed in approximately half of the patients with pigment, suggesting that other factors, such as diet habit changes or/and human genotype differences, might also influence the formation of pigment.

The presence of black extrinsic tooth staining has been reported to be associated with low caries occurrence in affected patients compared with individuals who have healthy tooth surfaces [40]. Even so, this study suggested that two plaque-community types may be separately associated with the low-frequency presence of CPP. Our results suggest that *Streptococcus* and *Mogibacterium* co-infection in the plaque of patients may be associated with the presence of mixed pigment and caries (Fig. 4c). *Streptococcus* species are commonly associated with dental caries [7, 35]. The cariogenicity of *Streptococci* in gnotobiotic rats is well established [47]. Moreover, *Mogibacterium* species have been linked to human periodontal diseases [48] and the acute dental abscess [49]. These findings raise the possibility that *Streptococcus* and *Mogibacterium* co-infections may be associated with clinical indicators of both periodontal diseases and the mixed occurrence of caries and pigment. Meanwhile, our results also revealed a significantly positive correlation among *Peptostreptococcus*, *Clostridium*, and unclassified *Clostridiaceae* in plaque, which may contribute to the presence of CPP in human populations with the type B community (Fig. 5b). *Peptostreptococcus spp.* is detected in primary endodontic infections [50]. The cariogenicity of *Peptostreptococci* in gnotobiotic rats is also well established [47]. The Clostridia are reported from odontogenic infections [49]. These findings suggest that those bacteria associated with plaque CPP may contribute to the occurrence of caries or other oral diseases, although there was no evidence of an association with the combination of pigment and caries.

## Conclusions

We propose the DMM approach is a good option to profile the human microbial communities associated with the human health or diseases based on 16S rRNA gene sequence analysis. Our results indicate a distinct microbial community and composition in the oral cavities of patients with caries, pigment, or a mixture of caries and pigment, suggesting that the microflora associated with a variety of oral diseases is heterogeneous. The plaque-associated flora is much more effective than those in saliva as biomarkers for diagnosing and/or preventing caries and pigment. This information can be used as a basis for understanding the involvement of specific bacteria in the occurrence of caries and pigment.

## Abbreviations

16S rRNA, 16S ribosomal RNA; ANOVA, one-way analysis of variance; CP, caries-active plaque; CPP, caries + pigment plaque; CPS, caries + pigment saliva; CS, caries-active saliva; DMM, dirichlet multinomial mixture; HOMOVA, homogeneity of molecular variance; HP, healthy plaque; HS, healthy saliva; NMDS, non-metric dimensional scaling; NMF, nonnegative matrix factorization; OTUs, operational taxonomic units; PAM, partitioning around meloids; PCoA, principal coordinate analysis; PP, pigment-occurred plaque; PS, pigment-occurred saliva

## Additional files

Additional file 1: Figure S1. Three typical cases from 40 patients with black-stained plaques. Y.HG.L., a specialist in pediatric dentistry, took responsibility for the black stain diagnosis and specimen collection. The clinical characteristics of all black-stained plaques as presented by three cases were softness and difficulty in removal. Supragingival black-stained plaque samples (including both the outside edge and the middle) from each patient with a typical black stain were pooled together for bacterial community analysis. (PPT 1583 kb) Additional file 2: Figure S2. Principal coordinate analysis (PCoA) of microbial communities based on (A) 111 salivary and 110 supragingival plaque samples and (B) 88 salivary and 80 supragingival plaque samples after sub-sampled to 2,000 reads. Both unweighted *Unifrac* and binary jaccard measures showed consistent patterns. The anosim test is used to detect significant difference between saliva and plaque (See Methods). (PDF 746 kb) Additional file 3: Figure S3. Rarefaction measures of bacterial 16S rRNA sequences from 111 saliva and 110 supragingival plaque samples. The rarefaction curve based on observed species counting under different sequence depth is plotted by sub-sampling from 100 to 2000 with 100 sequence increase each step without any replacement following ten replicates. (PDF 188 kb) Additional file 4: Figure S4. Community type assignment for non-metric dimensional scaling (NMDS) ordination of Bray-Curtis divergence values of 178 samples after subsampling using the DMM approach based on genus and OTU abundances. The stress computed for this ordination was 0.24, and the R^2^ between the input distance matrix and the distance matrix calculated between the points for this ordination was 0.81. (PDF 392 kb)
