# Supplementary material for: Oral microbial community typing of caries and pigment in primary dentition
Source: BMC Genomics. 2016 Aug 5;17:558. doi: 10.1186/s12864-016-2891-z (PMC4974685; doi:10.1186/s12864-016-2891-z)
Supplement: Additional file 1: Figure S1. — Three typical cases from 40 patients with black-stained plaques. Y.HG.L., a specialist in pediatric dentistry, took responsibility for the black stain diagnosis and specimen collection. The clinical characteristics of all black-stained plaques as presented by three cases were softness and difficulty in removal. Supragingival black-stained plaque samples (including both the outside edge and the middle) from each patient with a typical black stain were pooled together for bacterial community analysis. (PPT 1583 kb) [file 12864_2016_2891_MOESM1_ESM.ppt]

## Slide 1
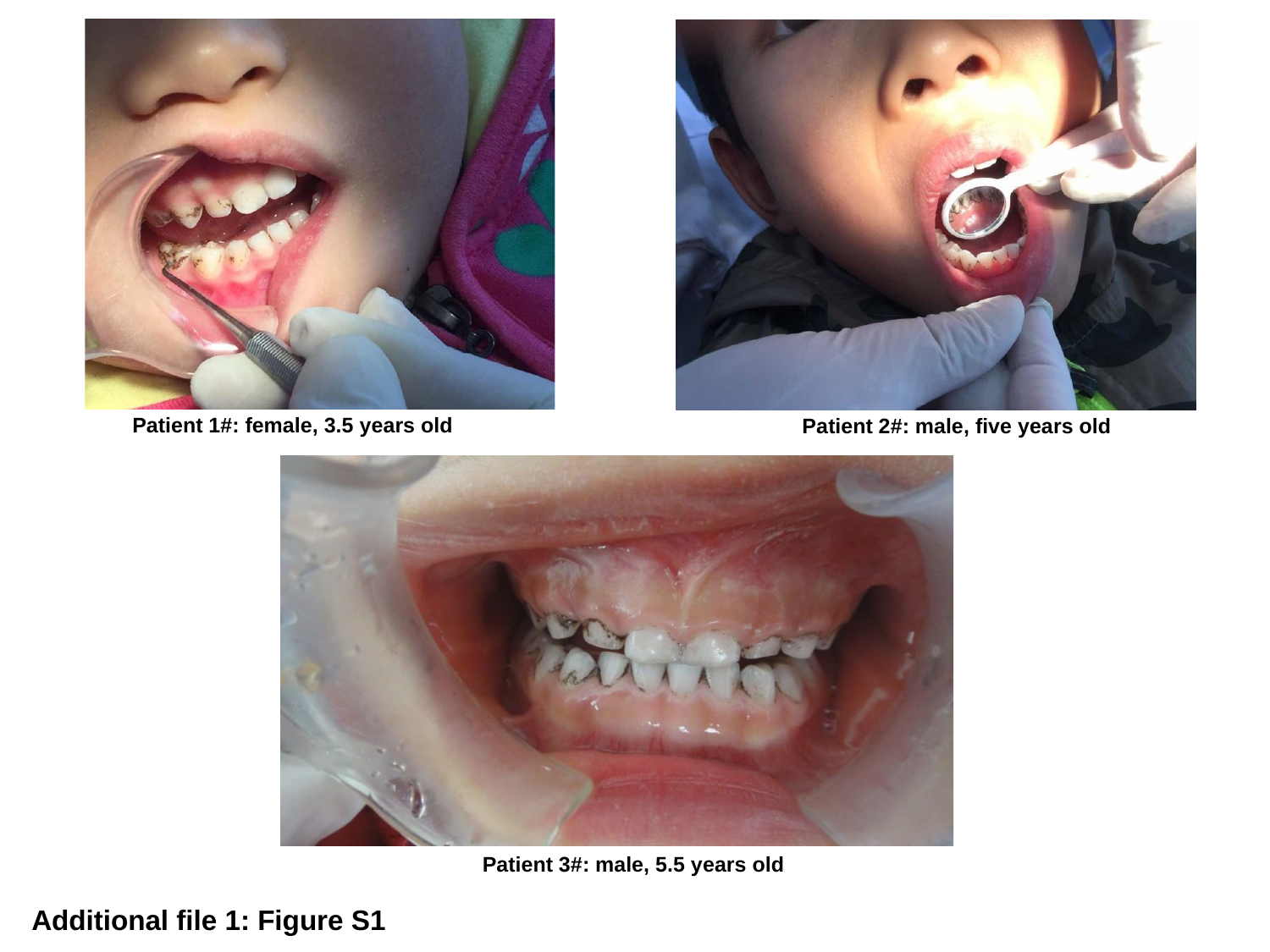

Patient 1#: female, 3.5 years old
Patient 2#: male, five years old
Patient 3#: male, 5.5 years old
Additional file 1: Figure S1
