# Supplementary figures and images for: Oral microbial community typing of caries and pigment in primary dentition
Source: BMC Genomics. 2016 Aug 5;17:558. doi: 10.1186/s12864-016-2891-z (PMC4974685; doi:10.1186/s12864-016-2891-z)

Additional file 2: Figure S2

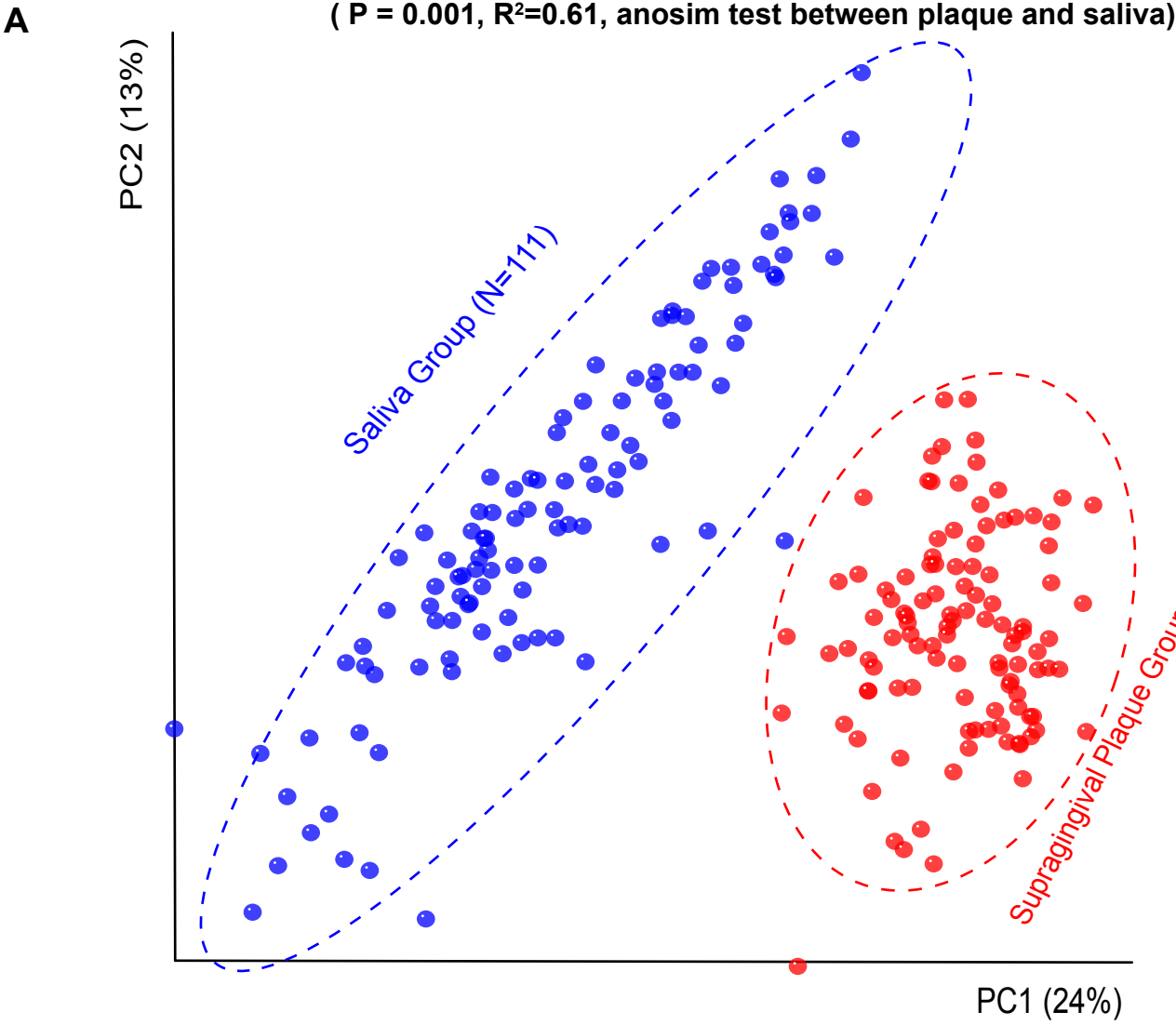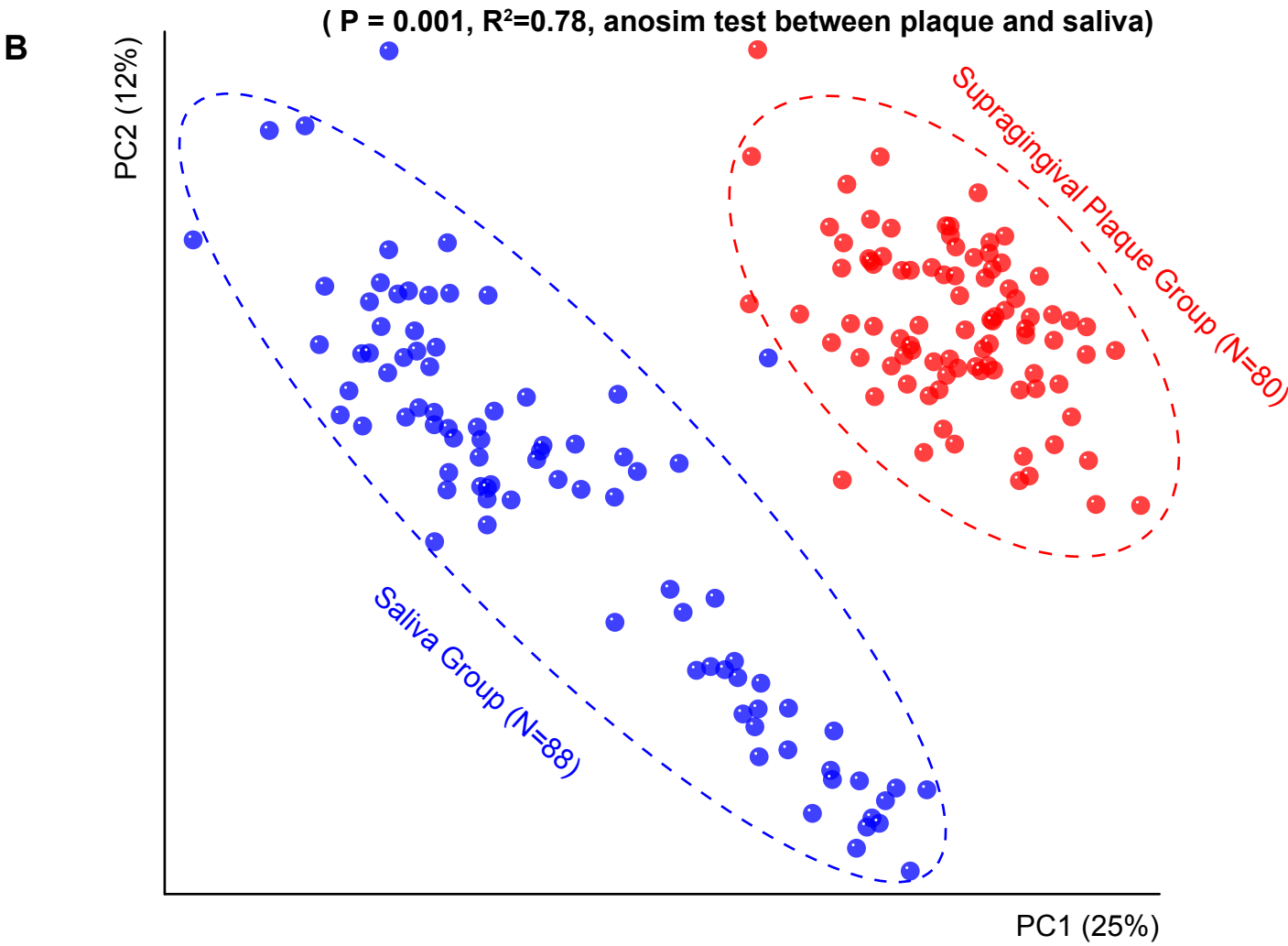

Supplement: Additional file 2: Figure S2. — Principal coordinate analysis (PCoA) of microbial communities based on (A) 111 salivary and 110 supragingival plaque samples and (B) 88 salivary and 80 supragingival plaque samples after sub-sampled to 2,000 reads. Both unweighted Unifrac and binary jaccard measures showed consistent patterns. The anosim test is used to detect significant difference between saliva and plaque (See Methods). (PDF 746 kb) [file 12864_2016_2891_MOESM2_ESM.pdf]

**Additional file 3: Figure S3**

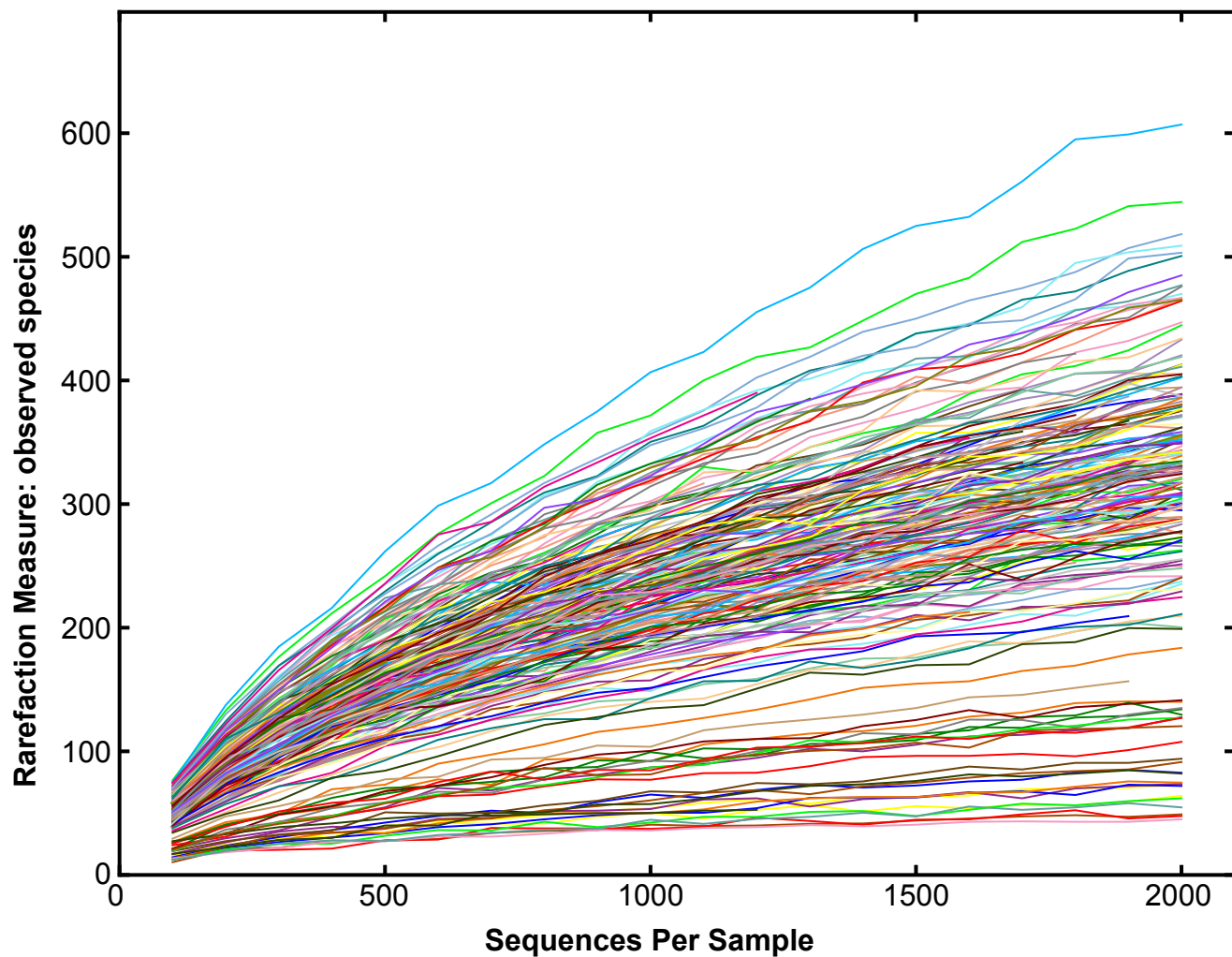

Supplement: Additional file 3: Figure S3. — Rarefaction measures of bacterial 16S rRNA sequences from 111 saliva and 110 supragingival plaque samples. The rarefaction curve based on observed species counting under different sequence depth is plotted by sub-sampling from 100 to 2000 with 100 sequence increase each step without any replacement following ten replicates. (PDF 188 kb) [file 12864_2016_2891_MOESM3_ESM.pdf]

Additional file 4: Figure S4

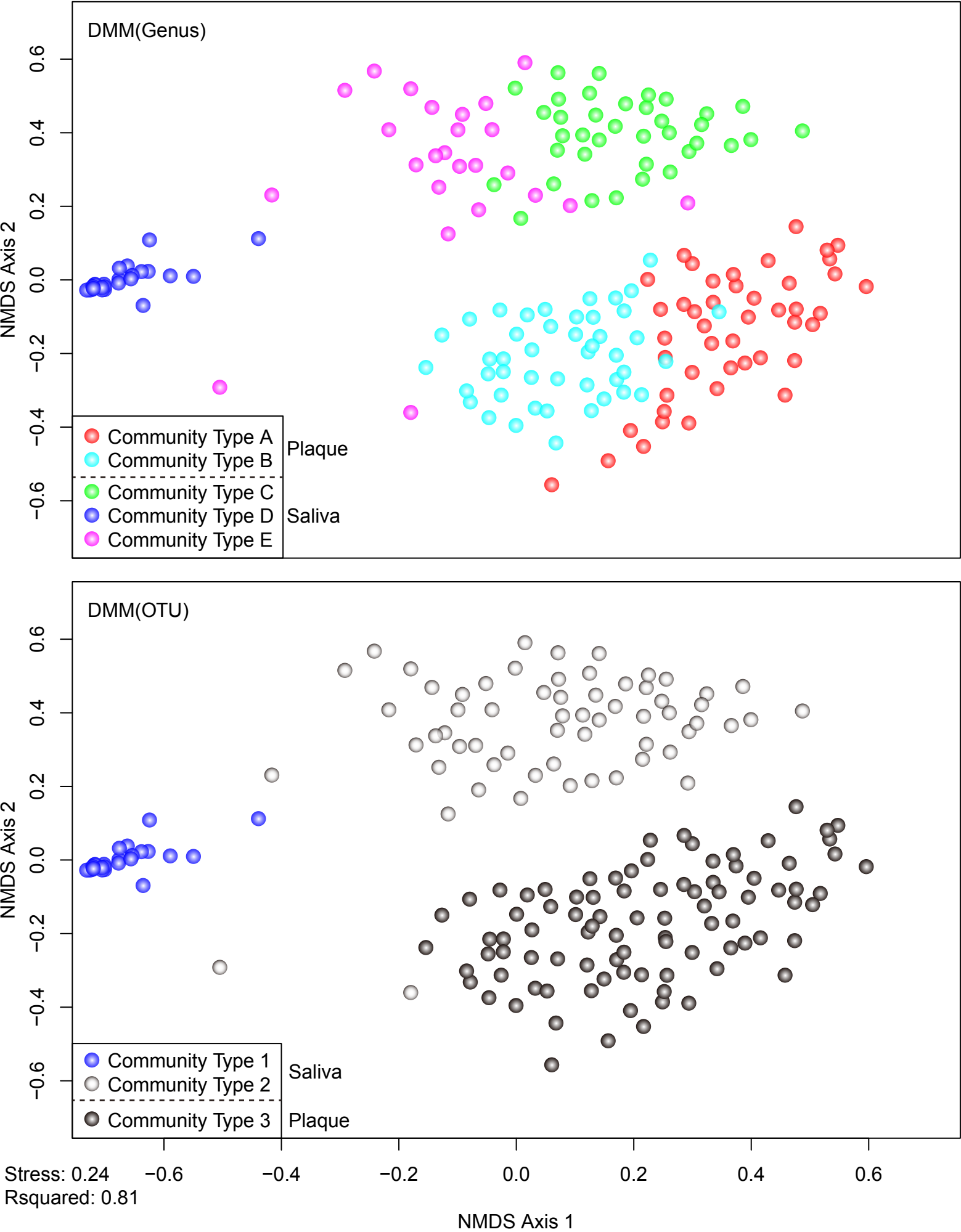

Supplement: Additional file 4: Figure S4. — Community type assignment for non-metric dimensional scaling (NMDS) ordination of Bray-Curtis divergence values of 178 samples after subsampling using the DMM approach based on genus and OTU abundances. The stress computed for this ordination was 0.24, and the R2 between the input distance matrix and the distance matrix calculated between the points for this ordination was 0.81. (PDF 392 kb) [file 12864_2016_2891_MOESM4_ESM.pdf]
